# The effect of chemotherapeutics on cell-to-cell transport of HTLV-1 and the p8 protein through membrane nanotubes

**DOI:** 10.1186/1742-4690-12-S1-P8

**Published:** 2015-08-28

**Authors:** M Omsland, C Pise-Masison, BT Gjertsen, G Franchini, V Andresen

**Affiliations:** 1 Centre for Cancer Biomarkers CCBIO, Department of Clinical Science, Translational Hemato-Oncology Group, University of Bergen, Bergen, Norway; 2 Animal Models and Retroviral Vaccine Section, Vaccine Branch, National Cancer Institute, National Institutes of Health, Bethesda, Maryland, USA; 3 Department of Internal Medicine, Hematology Section, Haukeland University Hospital, Bergen, Norway

## 

The tunneling nanotube (TNT) is a novel type of cell-to-cell communicator, 50-2 nm in diameter, F-actin containing structure connecting two or more cells. TNTs have been observed in a variety of cells to transport different components such as mitochondria, cell membrane components, multi-resistance genes and pathogens (like retroviruses and bacteria). The exact molecular mechanisms behind TNT formation are still unclear. HTLV-1 hijacks TNT-like structures for its transmission through the viral encoded p8 protein that augments the number and length of these TNT-like structures to favor virus transmission among T-cells. We have previously investigated TNTs in the heterogeneous and aggressive blood cancer, acute myeloid leukemia (AML), and found that the chemotherapeutic cytarabine (AraC) down-regulates TNT production in AML cells. Thus we wish to apply this knowledge to investigate whether AraC may exert a similar effect in T-cells and monocytes resulting in decreased HTLV-1 transmission. Furthermore we will measure the drug's effect on the transfer of the p8 protein to uninfected cells such as primary PBMCs, Jurkat T cells and THP-1 cells. We have generated Jurkat T and THP-1 cells stably expressing mem-GFP and mem-Cherry proteins for live-cell visualization of TNT and TNT-like structures by fluorescence microscopy. The results will be presented.

